# A Comprehensive Review of Friction Stir Additive Manufacturing (FSAM) of Non-Ferrous Alloys

**DOI:** 10.3390/ma16072723

**Published:** 2023-03-29

**Authors:** Adeel Hassan, Srinivasa Rao Pedapati, Mokhtar Awang, Imtiaz Ali Soomro

**Affiliations:** 1 Department of Mechanical Engineering, Universiti Teknologi PETRONAS, Seri Iskandar 32610, Perak Darul Ridzuan, Malaysia; 2 Department of Metallurgy and Materials Engineering, Mehran University of Engineering and Technology, Jamshoro 76062, Sindh, Pakistan

**Keywords:** metal additive manufacturing, friction stir additive manufacturing, solid-state, metallic laminates, grain refinement, non-ferrous alloys

## Abstract

Additive manufacturing is a key component of the fourth industrial revolution (IR4.0) that has received increased attention over the last three decades. Metal additive manufacturing is broadly classified into two types: melting-based additive manufacturing and solid-state additive manufacturing. Friction stir additive manufacturing (FSAM) is a subset of solid-state additive manufacturing that produces big area multi-layered components through plate addition fashion using the friction stir welding (FSW) concept. Because of the solid-state process in nature, the part produced has equiaxed grain structure, which leads to better mechanical properties with less residual stresses and solidification defects when compared to existing melting-based additive manufacturing processes. The current review article intends to highlight the working principle and previous research conducted by various research groups using FSAM as an emerging material synthesizing technique. The summary of affecting process parameters and defects claimed for different research materials is discussed in detail based on open access experimental data. Mechanical properties such as microhardness and tensile strength, as well as microstructural properties such as grain refinement and morphology, are summarized in comparison to the base material. Furthermore, the viability and potential application of FSAM, as well as its current academic research status with technology readiness level and future recommendations are discussed meticulously.

## 1. Introduction

**Figure 1:**
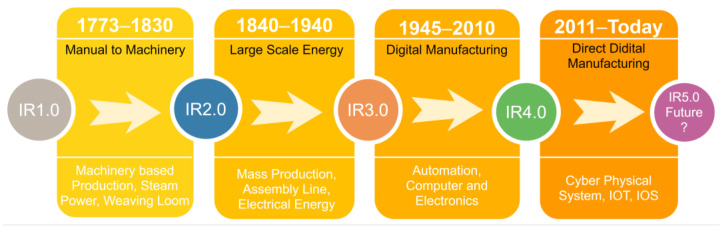
Chronological outline of industrial revolutions.

Prior to the industrial revolutions, agriculture, and handicrafts were the main drivers of economies. This trend was altered by the industrial revolution, which turned them into manufacturing-based economies. Industrial revolution is historically catalogued into four sessions illustrated in Figure 1. Before 1830, shifting of manual production to the machinery-based production was known as the first industrial revolution. The era of 1840–1940 is called second industry revolution, which involved the advancement of large-scale energy (electricity, petroleum) and material production. In the automobile and aircraft manufacturing industry, the production rate drastically increased to the mass production [1]. The third industrial revolution is named as digital manufacturing and it began in 1945. In this revolution, the technology moved from analog, mechanical, and electronic systems to the highly connected digital technology [2]. The fourth revolution is direct digital manufacturing and it was introduced by the German government in 2011 [3]. As a new phase, it embraces future technologies such as cyber systems, internet of things (IOT), the internet of services (IOS), robotics, big data, cloud manufacturing, and augmented reality, and has a great impact on the economy as well [4,5]. Currently, we are living in the fourth industrial revolution epoch which is commonly known as IR4.0.

**Figure 2:**
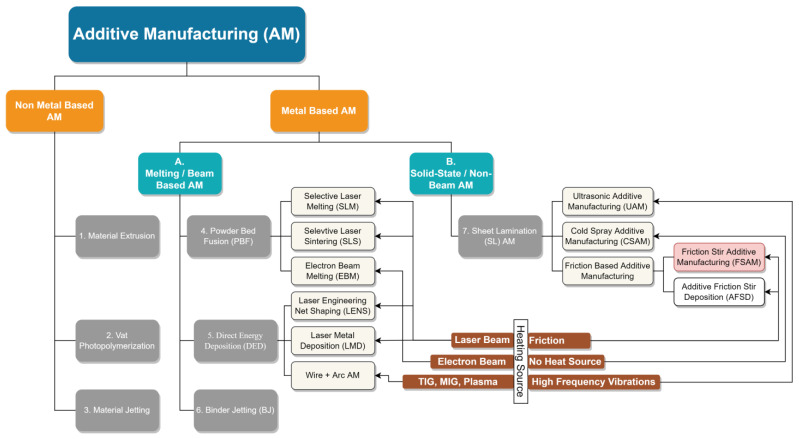
Family tree of current and future additive manufacturing processes-ASTM F2792.

Additive manufacturing (AM) is the vital part of the IR4.0, which is defined as to convert 3D CAD data to produce physical parts by joining material (metal, ceramic, or polymer) in layer-by-layer fashion [6]. This technology has been at the forefront from the last 30 years, and since from past one decade, it has entered in the mainstream industrialized field [7]. AM process is advantageous over conventional manufacturing such as low material waste [8], excellent part accuracy [9], less human commitment, and ecofriendly [10]. It is adopted in critical engineering fields such as the aerospace and automobile industry, but is still facing challenges to produce physical metallic components [11]. American standard for mechanical testing ASTM-F2792-12a grouped current and future AM technologies into seven families; a complete family tree of AM process is shown in Figure 2 [6]. The classification of AM techniques as per ASTM standard is: (i) binder jetting (BJ), (ii) direct energy deposition (DED), (iii) material extrusion (ME), (iv) material jetting (MJ), (v) powder bed fusion (PBF), (vi) sheet lamination (SL), (vii) and vat photopolymerization (VP). Broadly, material is classified into three classes such as metal, polymer, and ceramic, and AM technologies mainly depend on the class of candidate material [12]. Binder jetting is an AM technique of joining powder particles selectively by using a liquid-based binding agent. Metallic, ceramic, and polymer powder as feed materials are used in this process. Consequently, steel parts with excellent mechanical properties are produced with this process. No support structure is required; however, high level part shrinkage is the key challenge of this method [13]. Direct energy deposition additive manufacturing (DED-AM) is the process of fabrication physical part by depositing metallic powder or feed wire simultaneously in moving substrate under a vacuum or protective atmosphere of inert gas [14], and it is also used for metallic repair work [15]. In comparison to binder jetting, binder jetting yields better grain structure than DED-AM because of lower working temperature [16]. In material extrusion AM, the polymer or thermoplastic composites in wire or powder form as a feed material becomes softened and driven out through the orifice and is stacked to make a physical 3D standard component easily and cheaply as compared to the other AM processes [17,18]. Another commonly used AM technique in the field of polymer printing is material jetting AM. The droplets of build material are deposited and high-quality thin-walled featured parts with less staircase effect are produced, as compared to the other polymer printing process [19,20]. In powder bed fusion (PBF) AM, the build material in the form of powder pre-deposited on the bed is selectively diffused by high-source thermal energy to produce dense parts [6]. Since the past 20 years of progress, PBF-AM is still suffering from poor process repeatability [21,22] and lower deposition rate as compared to the DED-AM-AM [23]. Next, categorization is sheet lamination SL-AM, it is one of the most primitive commercialized AM processes and also known as lamination object manufacturing (LOM). The input material is cut into the desired shape, stacked, and bonded together to form a bulky objects, and the reuse of wrong pasted material is normally discarded [24]. Vat photopolymerization involves hardening of liquid resin that polymerizes when exposed to the light source of specific wavelength [25]. This technique is widely opted by the dental industry [26]. Among these classes, DED-AM, PBF-AM, SL-AM, and BJ-AM have promising potential for production of metallic functional parts of industrial applications [23]. From a variety of feed stock materials of current AM processes, if someone chooses the metallic material, only then AM processes arise into two factions such as melting or beam-based AM, and solid-state or non-beam-based AM.

### 1.1. Melting Based Additive Manufacturing

High energy laser/electron beam or electric arc is used to melt the feed metallic material (wire/powder pre-deposited on bed or feed through nozzle). Powder bed fusion (PBF) and direct energy deposition (DED) are the well-renowned beam-based additive manufacturing processes to produce metallic parts [27]. When high-energy beams interact with feed material, the complicated physical phenomenon of melting of feed material, flow of melting pool, and subsequently solidification occur [28,29]. Metal vaporization, excessive splashing, and larger heat affected zone (HAZ) involves when high energy beam comes into contact with feed material [30]. In the solidification phase: rapid cooling (103–107 Ks^−1^), due to the high thermal gradient and complex thermal cycles that might cause partial re-melting of the earlier deposited layer and epitaxial growth. Partial remelting of the already deposited layer plays a role of eradication agent of developed equiaxed grains on the top of the melt pool [31], which promotes textured columnar grain structure. The final mechanical and structural properties of produced parts are intensely affected by the microstructure/grain structure of the part. Most of the researchers reported that parts produced through existing fusion-based additive manufacturing techniques reveals anisotropic behavior and hence, non-uniform microstructure and inferior mechanical properties to the base material [11,30,32,33,34]. To encapsulate, the methodological framework of the current beam-based AM processes is melting and depositing one thin layer at a time, and it mimics micro-casting or micro-welding. Each melting based AM processes have their own set of advantages, such as good to reasonable surface finish, the ability to print complex geometries [35] with low material wastage, and flexibility in part customization [36]. However, some remarkable challenges, such as inability to process a wide range of non-weldable alloys (Aluminum 2xxx and 7xxx series), extremely high feed material costs, the presence of solidification defects (internal porosity and cavities [37], hot cracking and shrinkage [38], inclusions and high residual stresses [39] etc.) caused by liquid–solid phase transformation, low production volume, high operating costs, and less structural efficiency [33,40], currently limit the widespread acceptance of melting-based AM. Among these, priority of aerospace and automobile industries is high mechanical and structural efficiency with high production rate. So, these ongoing limitations could be overcome by adopting solid-state AM.

### 1.2. Solid-State Additive Manufacturing

To avoid the liquid-solid transformation defects, solid-state additive manufacturing is the substitute of existing beam-based AM. Being a solid-state process, there is no melting and no high-energy beam entailed, and input material is joined below its melting temperature. Consequently, solidification defects could be easily eliminated, which further harvests better microstructure along with improved mechanical properties. Ultrasonic additive manufacturing (UAM), cold spray additive manufacturing (CSAM) [41], friction stir additive manufacturing (FSAM), and additive friction stir deposition (AFSD) [42] are sub-classes of solid-state AM process.

Among these four well-recognized solid-state processes (UAM, CSAM, FSAM, and AFSD), friction stir additive manufacturing is a nascent technique, and hence relatively less work has been published yet. However, there is still a need to collect the data and review it to access the applicability of the currently evolving material synthesis technique in a better way. Hence, the goal of this paper is to explore FSAM by critically reviewing the process parameters and defects. Microstructure and mechanical characteristics, and technology readiness is explained as well. Google Scholar, Web of Science, ScienceDirect, and Scopus are the four main search engines being used to collect research articles published to date in English for this review paper.

## 2. Friction Stir Additive Manufacturing (FSAM)

**Figure 3:**
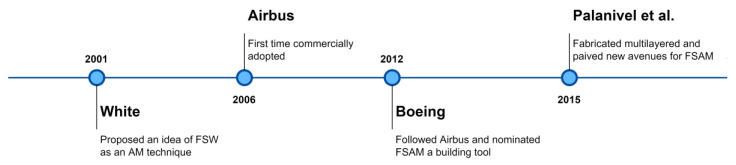
Timeline and development of FSAM [43,45,47,48].

Friction stir additive manufacturing is an emerging AM technique and falls in the category of sheet lamination AM (already discussed in the introduction para). Friction stir welding as an additive technique was first introduced by White [43] in 2002 by filing a patent. The proposed technique adopted commercially by Airbus in 2006 to produce wing ribs of Al-Li 2025 [44], and witnessed the excellent inter bonding of layers with more environmentally and greater production rate along with minimum material waste [45]. Unlikely, this could not gain much attention among the industrial and research community due to the lack of terminology and insufficient research, which hindered the further progress. Dilip et al. [46] used friction surfacing and friction welding as potential techniques for AM, and they called this friction stir deposition (FSD). Following that, Boeing in 2012 [47] evaluated the technique and nominated FSAM as a building tool for energy efficient structure developments. Curiously, after Airbus and Boeing’s works, there was no publication on FSAM until the work of Palanivel et al. [32,48] in 2015. Research works of Palanivel et al. have successfully created newer avenues in FSAM area. The timeline and development of FSAM is demonstrated in Figure 3.

**Figure 4:**
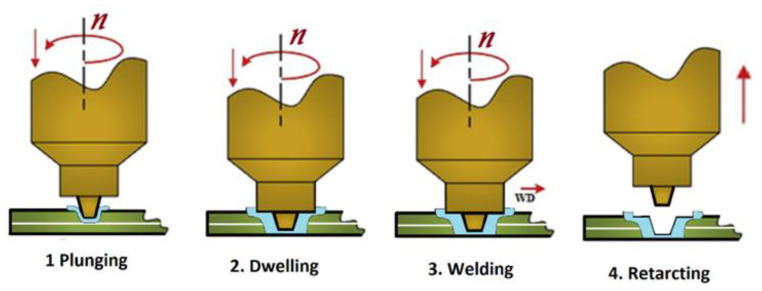
Stages involved in two layer joining (FSLW) (reprinted from [51] with permission from Elsevier).

**Figure 5:**
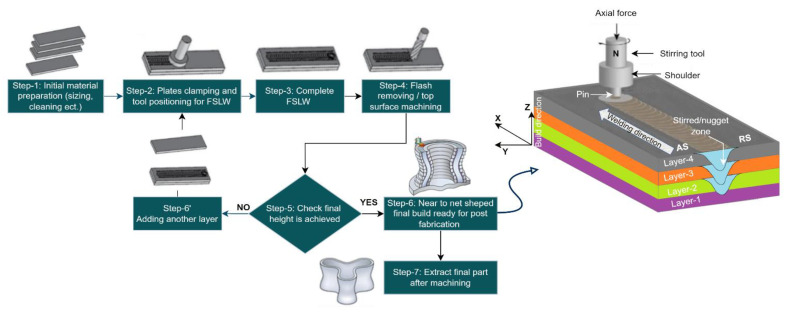
FSAM schematic illustration and final four-layered build obtained.

Basic working principal of FSAM is similar to the friction stir lap welding (FSLW), but the internal physic is slightly changed due to the addition of multiple laps step-by-step rather than once, which involves reheating and sintering [49,50]. The process of two layers addition via FSLW with single pass consists of four stages such as, plunging stage, dwelling stage, welding, and retracting stage, as shown in Figure 4. In the plunging stage, the non-consumable tool with constant rotation speed is plunged under axial force through the starting point until the tool shoulder touches the plates surface. The deformation process launches at this stage. In the dwelling stage, the rotating tool under axial force is dwelled for 5–10 s (depends upon the material nature and thickness) in time of contacting the shoulder to the surface to produce sufficient heat and plasticized workpiece. In the welding stage, the rotating tool that contains the plasticized volume beneath the shoulder travels along the second layer’s top joining line, which is known as the shoulder driven zone (SDZ). The plastic material agitated around the tool pin from advancing side (AS) to the retreating side (RS) in the “pin driven zone (PDZ)” at the bottom of the second layer and the top of the first layer. The shoulder forges the material behind the pin and fills the cavity effectively formed by the pin’s forward motion. Two layers successfully joined because of material intermixing, atomic diffusion due to the temperature and pressure. At the last stage of retracting, when the tool is reaching the end point of the weld, the tool is withdrawn from the deposited layer and leaves the layers to cool down [51]. The same steps are repeated until the desired height achieved. The build height depends on the thickness of each plate [30]. The complete FSAM method along with final build achieved is graphically demonstrated in Figure 5.

**Figure 6:**
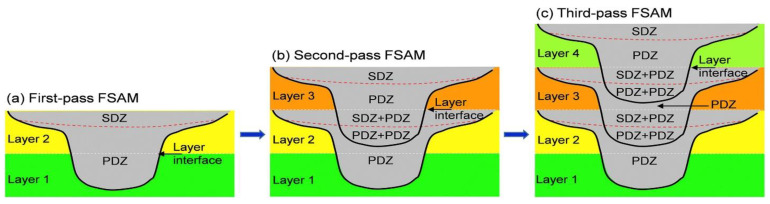
Schematic of stir zones of the FSAM build: (**a**) first pass FSAM; (**b**) second pass FSAM; and (**c**) third pass FSAM (reprinted from [50] with permission from Elsevier).

Since FSAM build consists of several lap joints, there would be more stir zones with greater degree of complexity. Complexity begins during the second pass of FSAM. Top of second layer is already shoulder and pin driven, thus when third layer will be added, the already existing SD and PD zones transform into SD + PDZ and PD + PD zones. The SD + PD region that denotes material flow is governed initially by the shoulder, and then by the pin. Similarly, the PDZ + PDZ zone indicates that this region of the material flow is governed by the tool pin twice. Same sequence of stir zone transformation is repeated until targeted height is not reached. Thus, different layers experience different thermal exposure from bottom to top layer of final build which leads to convoluted microstructural advancement of parts fabricated. These zones are graphically represented in Figure 6 [50].

To summarize the FSAM method, without the use of lasers, melting, or binders, the material is plasticized because of frictional heat. During processing, the peak temperature reaches in shoulder driven zone (SDZ) [50] and ranges between 60 and 90% of the melting point of feed material [52]. Parts with tailored microstructure and mechanical properties could be produced, but some sort of post processing in the form of machining or grinding is required [53]. Merits and some limitations of said manufacturing process are contrasted in Table 1.

## 3. Parameters Affecting FSAM

**Figure 7:**
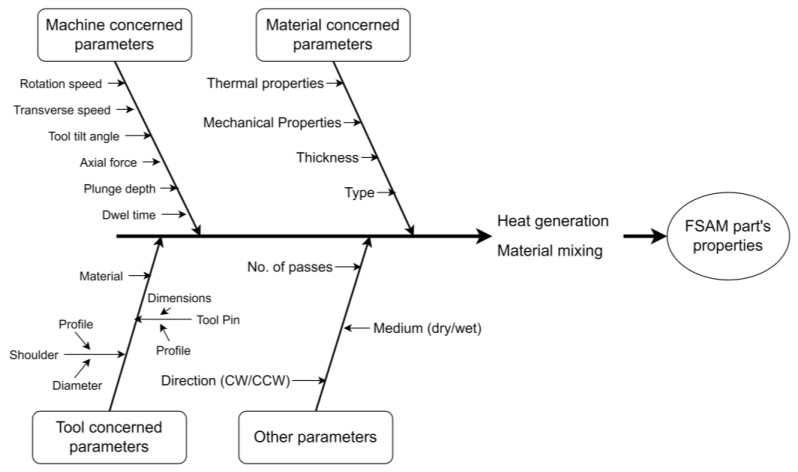
Factors affecting the microstructure and quality of the part produced through FSAM.

Main requirements of efficient FSAM process are sufficient heat production and better material mixing, which have greater influence over mechanical and microstructural properties of the final part [30,53]. The material mixing and heat generation is chiefly interlinked with the process parameters. Most of the FSAM processes parameters are analogous as those of FSLW/FSP, and categorized as machine concerned parameters, tool concerned parameters and material concerned parameters are elaborated in Figure 7. A comprehensive overview of parameters used by the various researchers are illustrated in Table 2 and Table 3 at the end of this section.

### 3.1. Machine Concerned Parameters

Machine parameters include rotation speed, transverse speed, tool post tilt angle, plunge depth, axial force, dwell time, and direction. Rotation speed and transverse speed are the two main heat-controlling input parameters. Rotation speed is in fact the friction speed between tool and plates, and rate of friction heat increases with increasing friction speed [30]. Transverse speed is a welded distance covered in a short period of time. Slow transverse speed causes high temperature in short displacement, whereas high transverse speed causes weaker stirring effect due to lessened heat generation [59]. Although both high rotation speed and slow transverse speed can increase the temperature, high rotation speed contributes more to temperature rise as compared to slow transverse speed. It is not always true that high rotation speed combined with low transverse speed results in defect-free parts having better mechanical properties. This combination may lead to excessive heat generation and some microstructural defects, as well as noticeable weld flash. On the other hand, simultaneous increment of both speeds can develop more residual stresses in the part which further affects its mechanical properties [60]. Tool post tilt angle is another important machine parameter for evaluating microstructure and mechanical properties of parts, which allows some of the tool shoulder to make contact with plate, while also producing a non-contact area. Tilted tool advocates better material mixing and heat generation as compared to zero tilt angle. When using the tilt angle, forging force increases, consuming more energy [61].

**Figure 8:**
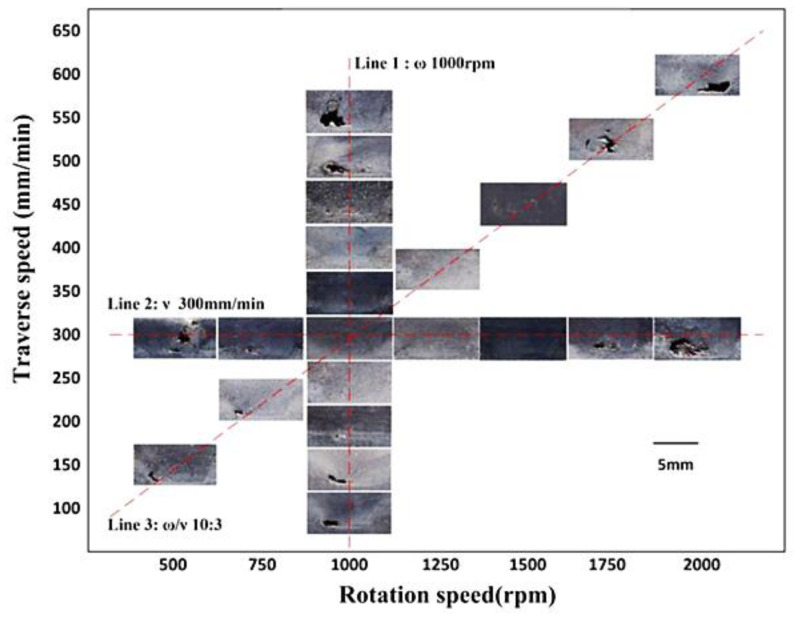
Rotation and transverse speed optimization by analyzing the major defects produced during FSLW of Al-2024 (reprinted from [63] permission not required).

S. Palanivel et al. [32] performed FSAM on 1.7 mm thick magnesium-based WE43 rolled condition sheets. Multi-layered build was achieved by using right-handed stepped spiral tool pin with 1.5° title angle at a constant transverse speed of 102 mm/min. Microstructure and defects were investigated under two different rotation speeds of 800 rpm and 1400 rpm. Higher rotation speeds (1400/120) produced defects due to the improper material mixing. Similarly, the effect of rotation speed on the formation of multilayer builds of aluminum lithium 2195-T8 alloy was explored by Z. Zhao et al. [62]. FSAM experimented under three rotation speeds of 800, 900, and 1000 and a constant transverse speed of 100 mm/min. The optimal microstructure was acquired at a lower rpm (800 rpm) rather than higher rpm. Both of these studies appealed that higher rotation speed does not always result in good material mixing, heat generation, and part strength. S. Zou et al. [63] stacked two layers of Al-2024 alloy together in lap configuration under varying welding speeds and rotation speeds. These two input parameters were optimized by analyzing the major defects after applying hit and trial approach; results are explained below in Figure 8. Experimental results exposed that plunge depth has minor effects of NZ quality when compared to the transverse and rotation speed. For good heat input, achievement at varying rotational (ω) and transverse (ν) speed, the ratio ω/ν should be kept 10:3. In single or multiple lap joints, the effective range of parameters is very small as compared to the butt joint configuration. Thus, it is very challenging to acquire the exact set of these parameters to obtain defect-free parts. Overall, summary of optimized set of machine parameters obtained by the researcher for defect-free built fabrication is depicted in the Table 4.

### 3.2. Tool Concerned Parameters

**Figure 9:**
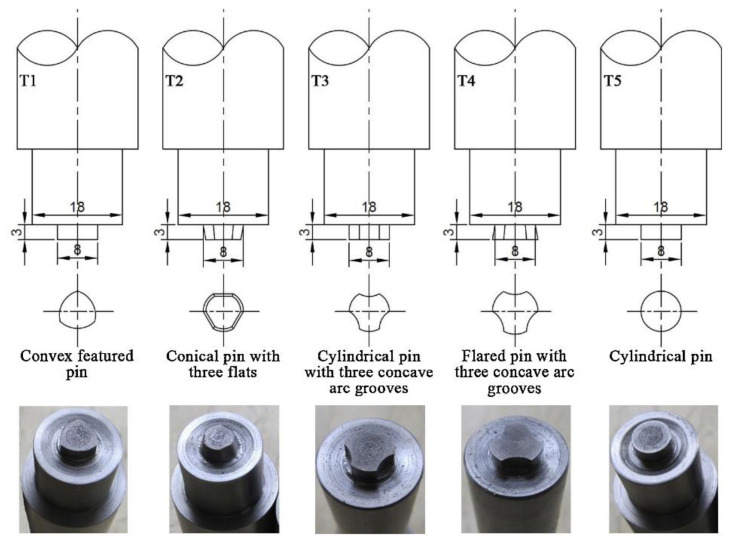
Different types of tool pin profile used to produce high quality Al-li 2195 build (reprinted from [62] with permission from Elsevier).

Tool concerned parameters are the pre-requisites of major machine-related parameters. Tool parts (shoulder and pin) geometry and material are the most significant tool concerned parameters. Tool shoulder geometry includes the diameter of the shoulder as well as whether it is concave or flat, whereas stirring pin geometry includes the type (square, cylindrical, conical, or threaded), length, and diameter. So, variations in these parameters will therefore affect the heat input, material mixing and flow, and thus the final microstructure of part. Tool shoulder generates 85% of the heat while the stirring pin produces the remaining 15% [64,65]. Heat creation is directly proportional to the shoulder contact, and it is determined from shoulder to pin diameter ratio (D/d). In the literature, it is reported that aluminum alloy exhibits sound mechanical properties at 3 ‘D/d’ ratio [66]. Regarding the pin profile selection, for obtaining the good microstructure, simply select the pin profile first and then tune the remaining parameters. It is a very long debate on pin profile; some reports claimed conical pin exhibits poor material mixing, so it is not suitable, another one claimed that the defect-free part can be produced by using the conical pin by tunning other parameters. To capsulate, all above mentioned parameters are interlinked with each other and we cannot ignore any single parameter. Thus, for FSAM, most of the researchers used cylindrical threaded and conical threaded pin, and then regulated the other parameters except Z. Zhao et al. [62]’s work. His research was pin profile-specific; five distinct pin profiles (T1: convex featured, T2: conical with 3 flats, T3: cylindrical with 3 arc grooves, T4: flared with 3 arc grooves, and T5: plain cylindrical) were chosen for the comparative analysis. Drawing and physical tool’s snapshot is displayed below in Figure 9. Experimental results disclose that tool T5 with plain cylindrical pin and tool T2 having conical pin with three flats yields unacceptable material mixing along the bonding interfaces. Meanwhile, tools T1, T3, and T4 result in good material mixing on the advancing side of NZ (with some microstructural defects), but not on the retreating side. Similarly, M. Sigl et al. [67] used double-scrolled stationary and rotating tool shoulder to perform FSAM, and announced the combined rotating and stationary tool shoulder produced defect-free Al-7075 structure.

### 3.3. Material Concerned Parameters

Material thickness, type, chemical composition, thermal, and mechanical properties are the material concerned parameters. Each material has its own set of thermal and mechanical properties that causes it to behave differently during the heat generation and material flow phases. Plate thickness determines the tool pin length and shoulder diameter (3.5 times plate-thickness) [68], which further facilitate to design tool pin accordingly. To the best of the author’s knowledge, the effect of variable thickness in the context of FSAM has not yet been practically investigated. Z. Zhang et al. [69] developed integrated models to explore the effect of variable layer thickness on the temperature and microstructure of the build. Three builds with layer thicknesses of 2 mm, 4 mm, and 6 mm, respectively, were investigated using finite element (FE) simulation. The results showed that increasing layer thickness decreases build temperature and average grain size while increasing strength and hardness.

**Table 2:** Overview of material concerned, and machine concerned parameters applied by the various researchers.

Sr. No.	Material Concerned Parameters		Machine Concerned Parameters					Ref.
	Material/No. of Layers	BH-mm	RS- rpm	TS-mmmin^−1^	PD *-mm	TA-Deg.	Medium	
1	WE43 rolled condition/4	5.6	800, 1400	102	—	1.5°	Air	[32]
2	AA5083-O solid sol. strength/4	11.2	500	152	—	1.5°	Air	[48]
3	AA 7075-O/9	42	600	60	0.2	2°	Air	[70]
4	2050 cast/12, AA2050-T3/7	432	250	204	—	1	Water spray	[71]
5	2195-T8 Al-Li/5	—	800, 900, 1000	100	—	—	Air	[62]
6	AA6061-T6/4, AA-6082(sub)	—	1000	100	—	—	Air	[49,69,72]
7	AA 7N01-T4/12	42	1200	60	—	—	Air	[73]
8	IF, St52 steel/2	—	600	40, 70, 100	—	3°	Air	[74]
9	PMMA, AISI 304/4 each	—	850	45	—	2.5°	TP ~280 °C	[75]
10	AZ31B-HA magnesium	—	900	30	—	3°	Air	[76,77]
11	AZ31-H24 mg alloy/7	—	1000	100	1.65	−0.5°	Air	[78]
12	2195-T8 Al-Li alloy/3	—	700	200	—	—	Air	[79]
13	A357/SiC AMMC and Al-6XXX/2	—	500, 1000	100, 200	1	3°	Air	[80]
14	Pure copper and steel/2	6	600	50	1–1.4	2°	Air	[81]
15	AA 6061-T651, Steel 1018/2	—	600, 1000	300,600	—	1°	Air	[82]
16	Al plates/4	—	800, 1000, 1200	100	—	—	Air	[83]
17	Al-7A04-T6/4	—	700	160	—	2.5°	Water 20 °C	[84]
18	7N01-T4/12	42	1200	80	—	—	Air/water 25 °C	[85]
19	AA6061-T6/4	—	1200	100	—	—	Air	[86]
20	Al–Zn–Mg–Cu sol. treated/4	—	700	160	—	—	Water 15 °C	[87]
21	Al–Zn–Mg–Cu 7A04-T6/4	10.5	700	160	—	—	Water 20 °C	[50]
22	PP and Textile SS/7	—	850	45	—	2.5°	TP ~180 °C	[88]
23	Pure Cu cold rolled T3	—	600	50	0.2	3°	Water	[89]
24	Al-Cu pipes AA5086 and C12200	—	400, 500, 600, 700	40, 60, 80	0.2	3°	Air	[90]
25	Al 5059-O/6	20	450	63	0.25	2°	Air	[91]
26	Al-5083-O. 6061-T6, 7075-T6/3	8.8	750	55	1.7	3°	Air	[92]
27	Al-7075-T6/5	—	2000	65, 80, 95	—	0.5°	Air	[67]
28	Al-6061, Al-7075/7	—	1200, 1100	40, 50	1.15	2°	Air	[93]
29	Al-5083, Al-7075/3	—	850	55	—	—	Air	[94]
30	Al-2060/2	4	1500–1800	300–500	—	—	Air	[95]
31	Mg-AZ91, Cu, Al-7075/3	—	2000	40	—	0	Air	[96]

Legend: BH—build height; RS—rotation speed; TS—transverse speed; PD *—plunge depth; TA—tilt angle; TP—tool pre-heated.

**Table 3:** Overview of tool concerned parameters applied by the various researchers.

Sr. No.	Tool Concerned Parameters				Ref.
	Pin Profile/Length-mm	PD-mm d1/d2	SD-mm	Material	
1	Right-handed stepped spiral/2.2	3.5/6	11.8	Tool steel	[32]
2	Triple flat left-handed stepped spiral tool/4.75	3.9/5.9	10	—	[48]
3	Left cylindrical threaded pin/5.2	14/14	30	GH4169 steel	[70]
4	Threaded taper with 3 flats/12.85	8.3/12.7	28.6	—	[71]
5	- Convex featured and conical with 3 flats - Cylindrical and flared with 3 concave arc grooves - Plain cylindrical/3 mm	8/8	18	—	[62]
6	Conical Pin/8	6/8	24	H13 steel	[49,69,72]
7	Right-handed stepped spiral/5	5/6	15	Tool steel	[73]
8	Cylindrical/0.5	6/6	20	WC	[74]
9	Threaded corner-removed triangle with hole/6	6/6	20	—	[75]
10	Cylindrical treaded/6	6/6	16	Nitrated HSS	[76,77]
11	Threaded taper triangular/6.5	4.5/7	18	H13 steel	[78]
12	—	—	—	—	[79]
13	—	—	—	—	[80]
14	Plain taper/3.1	3/5	10	WRe	[81]
15	Cylindrical/6, 6.2	8/8	18	H13 steel	[82]
16	—	—	—	—	[83]
17	—	—	—	—	[84]
18	Conical threaded/5	5/6	15	—	[85]
19	Conical/7	6/7	24	—	[86]
20	Conical threaded/4	5/7	15.5	—	[87]
21	Conical threaded/5.5	4.2/7	15.5	—	[50]
22	Threaded corner-removed triangle with hole/6	6/6	20	—	[88]
23	Tapper threaded/2.1	4.4	10	—	[89]
24	Cylindrical/2	3/3	10	—	[90]
25	Tapper threaded/5	4/6	12	H13 steel	[91]
26	Tapper threaded/4.7	3/7	25	H13 steel	[92]
27	Threaded taper with 3 flats/6	8	20	—	[67]
28	Tapper threaded/4	6/8	24	H13 steel	[93]
29	Tapper threaded/4.7	4.7/7	25	H13 steel	[94]
30	Conical/3	3/5	10	—	[95]
31	Cylindrical threaded	4	11.8	HSS	[96]

Legend: PD—pin dia; d1/d2—tip dia/root dia; SD—shoulder dia; mm—millimeters; HSS—high speed steel.

**Table 4:** Summary of material and optimized set of parameters for successful defect-free layered build fabricated to date.

Material	Parameters					Ref.
	Pin Profile	rpm	mm/min	Tilt Angle	Medium	
AA5083-O	Triple flat left-handed stepped spiral	500	152	1.5°	Air	[48]
PMMA, S304 AISI	Threaded corner-removed triangle	850	45	2.5°	Air, TP ~280 °C	[75]
Mg alloy AZ31-H24	Threaded taper triangular	1000	100	−0.5°	Air	[78]
A357/SiC AMMC and Al6XXX	—	500, 1000	100, 200	3°	Air	[80]
Pure copper and steel	Plain tapper	600	50	2°	Air	[81]
Al–Zn–Mg–Cu 7A04-T6	—	700	160	2.5	water	[84]
7N01-T4	Conical threaded	1200	80	—	Air/water	[85]
AA6061-T6	Plain conical	1200	100	—	Air	[86]
Al–Zn–Mg–Cu	Conical threaded	700	160	—	water	[87]
Al–Zn–Mg–Cu 7A04-T6	Conical threaded	700	160	—	water	[50]
PP and Textile SS	Threaded corner-removed triangle	850	45	2.5°	Air, TP ~280 °C	[88]
Pure Cu cold rolled T3	Tapper threaded	600	50	3°	Water	[89]
Al-5083, Al-7075	Tapper threaded	850	55	—	Air	[94]

## 4. Defects Elicited in FSAM

As discussed earlier, the FSAM is tantamount to the friction stir lap welding, so the defects produced during this process are also akin to the most common FSLW defects, such as hook formation, weak/kiss bonding, void, cavity, cracks, and tunnel. The key reason behind these defects are the improper selection of machine-related parameters such as rotation and transverse speed and tool geometry, as well which advocates the deprivation of build’s mechanical properties [73].

### 4.1. Hook and Kiss Bonding Defect

Hook defect is basically unbonded interface which is formed in the material flow direction means from advancing side (AS) to retreating side (RS) due to the selection of inadequate combination of rotation and transverse speed with tilt angle, and excessive plunge depth [97,98]. Similarly, weak or kiss bonding defect in aluminum alloy fabrication through friction welding is majorly caused by existing excessive oxide and impurities at the lap interface, which restrict sufficient material stirring and occur only in the retreating side (RS) of weld [99,100].

**Figure 10:**
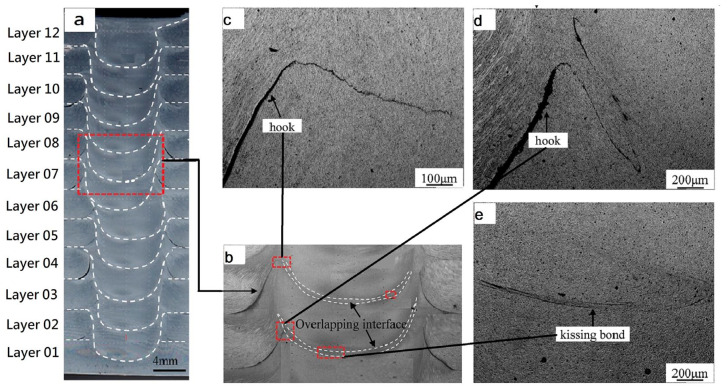
Hooking and kiss bonding defect in the identical material 7N01-T4 aluminum alloy build fabricated through FSAM (reprinted from [73] with permission from Elsevier): (**a**) laminate cross-section; (**b**) magnified layer six, seven and eight; (**c**) magnified hook defect of layer seven and eight; (**d**) magnified hook defect of layer six; (**e**) magnified kissing bond defect of layer six and seven.

Yuqing et al. [70] stacked nine layers of AA7075-O through FSAM by opting a set of parameters such as 600 rpm, and constant transverse speed of 60 mm/min with a 2° tool tilt angle. They explored the hook and kiss bonding defect in the final build and reported that hook sweeps in the nugget zone on AS, and moves upward on RS during first lap. After adding a third layer (second lap), the plasticized material of new added layer when extruded, it bends the hook upward which limits it to insert in the nugget zone. Kiss bonding defect was also observed due to crumbling of oxide layer which leads to the insufficient material flow. Similarly, C. He et al. [73] achieved a 42 mm tall build of 7N01-T4 aluminum alloy consisting of 12 layers at 1200 rpm and 60 mm/min speed. Hook and kissing defects were noticed in the seventh and eighth layer is shown in Figure 10. Kissing defects form due to the oxidation layer formation in two adjacent layers and dispersed irregularly along travelling direction, which can be avoided by increasing forging force. Similarly, the same type of defects were observed by H. Venkit [93] during fabrication of alternative gradient composite structure containing multiple sheets of Al-6061 and Al-7075. M. Sigl et al. [67] used FSAM to improve weld top surface and quality (micro-defects). The study’s main goal was to reduce machining costs while maintaining maximum and gap-free building height by adding the next plate without machining or milling. Two sets of builds were produced with rotating shoulder and rotating + stationary shoulder with their own set of parameters. Hooking defects were observed in each layer, and a flawless four layered build without machining was successfully produced with combined rotating and stationary tool shoulder. It is suggested that every fourth layer be machined to yield a low production cost.

To incapsulate, these defects can be minimized by considering the other parameters such as tile angle, plunge depth, and number of passes. Thus, Z. Zhao et al. [62] and S. Zou [63] used the double pass (back and forth) approach without the application of title angle to join 2 mm thick 2195T8 aluminum-lithium alloy plates and Al 2024-O, 5 mm thick plates, respectively, and successfully removed the hooking and kissing bonding defects.

### 4.2. Tunnel, Micro-Voids, Pores, and Cracking Defect

**Figure 11:**
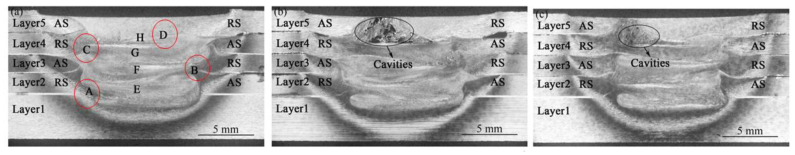
Al-li 2195-T8 build fabricated with single pass and flared tool pin having three concave arc grooves: (**a**) build fabricated at 800 rpm; (**b**) build fabricated at 900 rpm; (**c**) build fabricated at 1000 rpm. (reprinted from [62] with permission from Elsevier).

**Figure 12:**
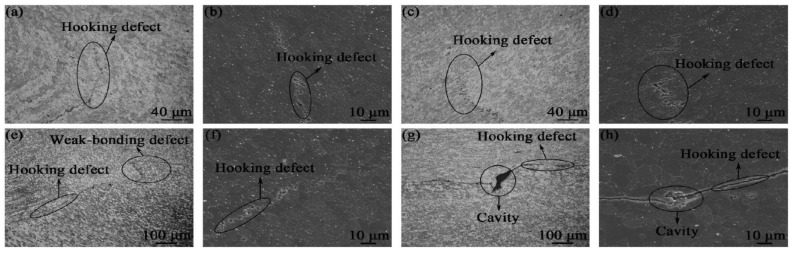
Hooking and cavity defects illustration of Al-li 2195-T8 build fabricated at 800 rpm with single pass and flared pin: Four regions of (**a**,**b**) A, (**c**,**d**) B, (**e**,**f**) C**,** and (**g**,**h**) D (reprinted from [62] with permission from Elsevier).

Tunnel or cavity is basically a missing piece of plasticized material owing to the inadequate heat generation and pressure, small tool tilt angle, and plunge depth [101,102,103,104]. At very high rotational speeds, the material does not rotate in a regular manner around the pin, due to the non-steady temperature distribution causing abnormal stirring, which is another cause of cavity formation [97,105,106]. Generally, these defects form at close to the bottom of lap in the advancing side (AS) when material propelled from retreating side (RS) with the help of tool pin [107,108]. Larger intermetallic compounds IMCs layer formation in the nugget zone may also be the option which yields tunnel or cavity and is usually observed in dissimilar lapping, owing to the different thermal properties [105]. Existence of these defects further facilities the cracking defect. Higher forging force is required to provide necessary compaction pressure to fill or close the cavity. S. Palanivel et al. [32] fabricated builds consisting of four layers of magnesium-based WE43 alloy-based alloy and found a large number of cavities, and some other defects such as cracking at a rotation speed and transverse speed of 1400 rpm and 102 mm/min, respectively. Similarly, S. Wlodarski et al. [78] synthesized a seven-layered build of magnesium alloy AZ31 using tapper threaded tool pin and, during the process parametric optimization stage, noticed the cavity/worm hole and tunnel defects when tool rotation was less than 1000 rmp and transverse speed was greater than 100 mm/min. Veerendra Chitturi [109] also encountered tunnel and micro-void defects during aluminum and steel FSLW, which he eliminated by increasing the tilt angle to 2.5. Micro-voids were reduced correspondingly by increasing plunge depth by increasing pin length from 4 mm to 4.3 mm. Similar defects were also identified by Z. Zhao et al. [62] in their research, and these defects were further eliminated by applying double pass technique. Defects produced prior to the application of the double pass are depicted in Figure 11 and Figure 12. Many other researchers claimed these most common defects, so a summary of defects noticed by researchers is illuminated in the Table 5, along with material synthesized and selected parameters.

## 5. Microstructure and Mechanical Properties of FSAM Build

The suitability of an engineering product for a specific application is determined by its mechanical properties such as strength, hardness, elongation/ductility, and fracture toughness after fabrication. Hardness is related to strength [110] whereas, designers recommend a minimum of 5% ductility [111]. These properties involve reaction to applied load, which determines the service life of the part [100], which is related to the microstructure and grain morphology. To avoid disaster, the minimum requirements recommended by the design engineer must be met. So, this section reviews the microstructure and mechanical properties of identical and multi-material laminates. Mechanical properties compared with base materials and grain size of synthesized materials are graphically illuminated at the end of each section.

### 5.1. Identical Material Laminates

#### 5.1.1. Magnesium Based Alloys

S. Palanivel et al. (2015-a) [32] studied the structural efficiency of magnesium (Mg-4Y-3Nd) alloy using FSAM. After FSAM, the hardness improved whereas strength reduced, which further improved to 135 HV and 400 MPa, respectively, after aging (180 °C/60 h) compared to the base material. Ductility increased ten times in the final build but decreased after aging, but was still higher than the base material. The build fabricated at a higher rotation speed of 1400 rpm showed non-uniform hardness. The grain size of the material was found to be finer (2–3 µ) as a result of dynamic recrystallization. Similarly, S. Wlodarski et al. [78] fabricated a seven-layer magnesium alloy AZ31 build to produce large areas without volumetric defects using three parallel welding passes. Grain refinement and mechanical properties, such as strength and hardness, as well as fatigue strength, were investigated. Overall, % elongation, monotonic tensile, and fatigue strength were found to be lower in fabricated builds than wrought Mg-AZ31-O alloy due to cold work strengthening loss. Both hardness and strength showed a non-homogeneous trend in the building direction, resulting in a reduction in fatigue performance, which could be improved further by applying appropriate post-heat treatment.

#### 5.1.2. Aluminum Based Alloys

Later, in S. Palanivel et al., 2015-b [48], aluminum alloy Al-5083 four sheets were synthesized at 500 rpm with 152 mm/min transverse speed through FSAM and 18% (104 HV), and increased hardness was found in the build compared with parent material (88 HV) due to the dynamic recrystallization which yields finer grain size. Both yield and ultimate tensile strength improves to 267 MPa, 362 MPa, respectively, as compared to the base material (190 MPa, 336 MPa), respectively. Z. Zhao et al. [62] performed FSAM on 2 mm thick Al-Li 2195-T8 plates and reported inhomogeneous hardness and ductility results. Similar trend of inhomogeneity of hardness was also disclosed by M. Srivastava [91] while fabrication of Al-5059 through FSAM. From the first to the fifth layer, the hardness value and elongation varied between 95.7 and 116.8 HV, and 9.6 and 16%, respectively. Tensile strength will gradually decrease as the number of layers increases. The strength on the fifth layer (tope layer) was 272 MPa, while the strength on the first lap (2nd layer) was 348 MPa, representing 56.6% of the strength of the base Al-Li 2195 material (615 MPa). Recently, Z. Shen et al. [79] further explored the same Al-Li alloy (Al-2195) by elucidating microstructure evolution, and found that hardness in all three zones (AS, RS, NZ) were lower compared to the base metal due to precipitate dissolution, and a higher degree of softness due to increased heat input. Maximum strength of 399 MPa and an elongation of 8.4% in the longitudinal direction at nugget zone. The characteristics of precipitated particle distribution cause results to vary. EBSD analysis revealed that the grain size decreased from top to middle and middle to bottom in the order of 3.2 μm, 2.6 μm, and 2.0 μm, respectively. Whereas, the fraction of high angle boundaries (HABs) increased from top to bottom. At the bottom, grain types were recrystallized (43.98%), sub-structured (36.87%), and deformed (19.14%), whereas at the top, recrystallized type grains are less (24.094%), and both sub-structured and de-formed types are nearly equal (38%). Because the pinning effect of dislocation severely limits the development of recrystallized grains.

Zhao Zhang et al. [69] researched the effect of variable layer thickness on the microhardness and strength of Al-6061-T6 numerically and experimentally. Three sets of builds were created, each with multiple layers of thickness 2 mm, 4 mm, and 6 mm. They reported that hardness gradually rises from the bottom to the top layer in every build, with a maximum hardness of 104.3 HV (approximately equal to the BM). Both hardness and strength have a direct relationship with layer thickness; as layer thickness increases, hardness and strength will also increase. In the earlier study of Zhao Zhang et al. [49], the effect of re-stirring and re-heating on mechanical properties of same material (Al-6061-T8) were analyzed, and claimed the same trend of hardness and strength rise (bottom to time). Re-stirring and re-heating produced higher hardness and strength due to the drop of peak temperature.

Multi-layered build comprising twelve sheets of Al-7N01-T4 was developed in air by C. He [73] to study the aging effect on microhardness and tensile properties, as well as microstructure evolution. Samples were undergone for natural aging of 5, 30, 60, 90, 180 days, and artificial aging of 120 °C for 24 h. Hardness was measured in the build direction, and it was found that the grain size was going to be coarser from top to bottom, which contributed to a decrease in hardness steadily form top layer to the bottom layer with some inhomogeneous results. The same trend was also reported by M. Yuqing et al. [70]. Hardness increased as aging time increased up to the 60 day and after that, there was no noticeable difference in the hardness, which resembles with G. Zheng’s results [112]. Ultimate tensile strength increases with an increase in aging time whereas, elongation does not change as much after 5 days of aging. Overall, hardness and strength decreased with non-uniform trend in the build as compared to the base material, which cannot be recovered by any aging process (natural or artificial). Same results were reported by other researchers in their respective studies [70,113,114,115]. The factors behind these problems are macroscale softening, which is grain growth, and coarser precipitate with decreased dissolution by thermal cycling and static annealing. Microscopy and EBSD were used to characterize the grains in the laminate’s top, middle, middle-overlapping interfaces, and bottom regions. The average grain sizes grew in the following order: overlapping interface (2.48 μm), top (2.86 mm), middle (3.02 μm), and bottom (3.30 μm). Reinforced cooling was found to be an effective method of controlling and optimizing microstructures. So, in the continuation of this work, Y. Li et al. [87] studied the post-aging effect on Al-Zn-Mg-Cu alloy built under water. After underwater FSAM, the samples were aged for 7 days naturally (NA-7d) and artificially (AA) for 24, 48, and 72 h at three different temperatures (80 °C, 100 °C, and 120 °C). Over-aging was observed at 120 °C in the low heat affected zone (LHZ), which was resolved by lowering the ageing temperature to 100 °C. This resulted in a maximum hardness of 178 HV and ultimate tensile strength of 532 MPa in the high heat affected zone (HHZ) when artificially aged for 48 h.

Most of the researchers claimed overall inferior hardness and strength to the base metal, as well as non-homogeneous results. Thus, J. Li et al. [86] improved the mechanical properties of Al 6061-T6 alloy by analyzing the chemical composition of precipitates instead of reinforcing powder or any aging application. They concluded that hardness and strength of aluminum 6xxx series alloy can be improved by increasing precipitate quantity. When silicon concentration is too high, increasing the silicon content can increase the silicon content in the solid solution, but not the number of precipitates. The result of increase solid solution leads to an increase in hardness and yield stress. When the silicon content is too high, the volume fraction can be increased by increasing the magnesium content. The average grain size in the stir zone can be reduced as the volume fraction increases. The hardness and yield strength can be artificially controlled to increase along the additive direction when the weight percentage of magnesium remains constant, and the weight percentage of silicon of each additive layer increases along the additive direction.

**Figure 13:**
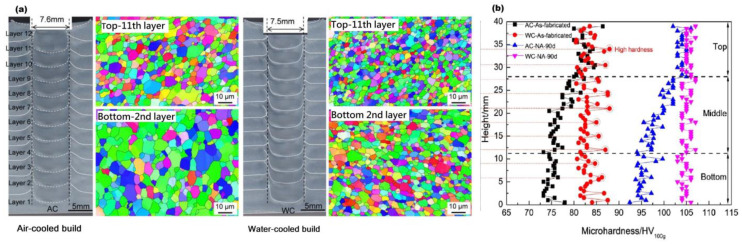
Natural aging and under water fabrication effect on microstructure and hardness of identical material laminates: (**a**) Microstructure comparison; (**b**) microhardness comparison (reprinted from [85] with permission from Elsevier).

**Figure 14:**
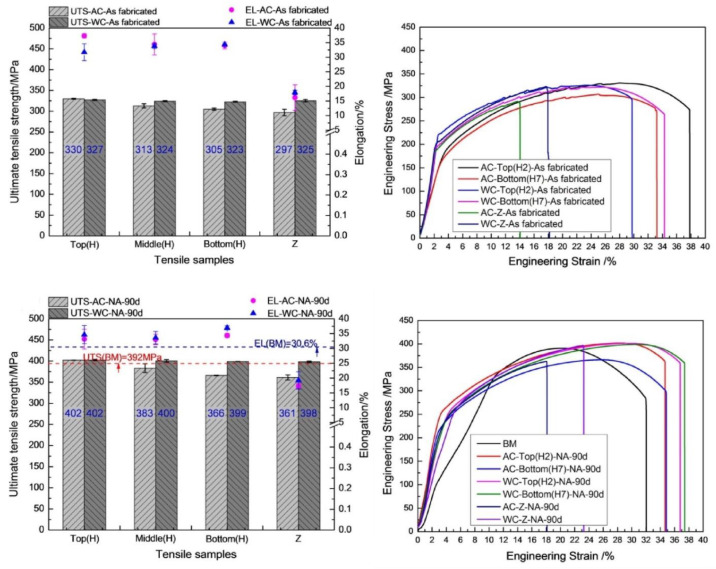
Tensile strength comparison of air-cooled and water-cooled identical material builds (at the top and bottom), as well as natural aging application on as-fabricated laminates (reprinted from [85] with permission from Elsevier).

Similarly, C. He et al. [85] suppress the common problem of variable and inhomogeneous hardness and strength by conducting the experiments in a water bath. In this manner (underwater), macroscale softening was successfully controlled, resulting in more uniform hardness, strength, and elongation. The results compared with air cooled build are illustrated below in Figure 13 and Figure 14.

**Figure 15:**
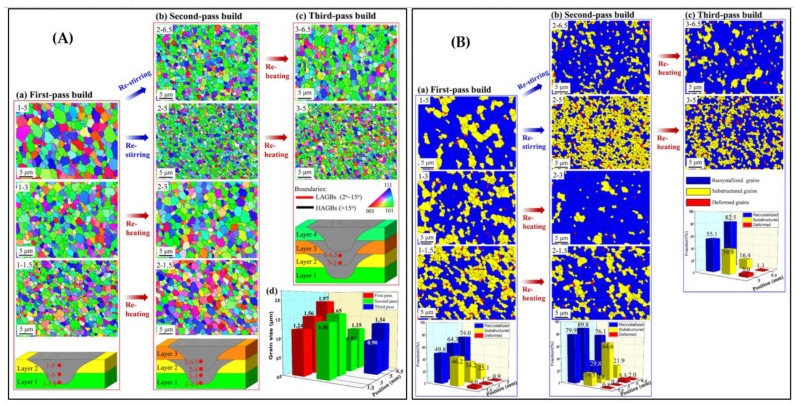
Effect of re-stirring and re-heating on grain characteristics of Al-7A04-T6 build: (**A**) Stir zone and distributions of grain sizes; (**B**) Fraction of grain type (re-crystalized, sub-structured, and deformed). (reprinted from [84] permission not required).

Effect of re-stirring and re-heating (under water) on microstructure evolution for Al-Zn-Mg-Cu alloy (Al-7A04-T6) was well explored in a recent study [84]. Four plates were laminated, and the samples were subjected to electron backscatter diffraction (EBSD) for grain morphology investigation after each pass (first, second, and third). During the single pass, the grain size and degree of recrystallization decreased from top to bottom. After restirring during the subsequent process, the grain size at the bottom of the over-lapping region decreased from 1.97 µm to 0.87 µm, while the recrystallization degree decreased from 74.0% to 29.8%. The grain size and recrystallization degree in the regions near the new additive zone increased slightly after reheating. Furthermore, there is no discernible effect of reheating on the microstructures of the regions. After reheating, the grain size and recrystallization degree in the regions close to the new additive zone increased slightly, but the local texture and precipitation remained unchanged. Reheating had no discernible effect on the microstructure of the regions. Figure 15 depicts conclusively the grain size and grain type (re-crystalized, sub-structured, and deformed) at various stages.

#### 5.1.3. Copper Based Alloys

M. Liu [89] followed the underwater approach and lapped 2 mm thick five pure copper plates. With optimized parameters of 600 rpm, 50 mm/min transverse speed, 3-degree tool tilt angle, and 0.2 mm tool shoulder plunge depth, eight passes were performed on each layer with threaded cylindrical tool pin to produce large areas. Superior strength and microhardness were observed, with a homogeneous trend than base metal. Maximum strength was 456 MPa (BM-271 MPa) and hardness was 141.3 HV (BM-130.2 HV). After EBSD characterization, the inverse pole figure (IPF) maps show that the equiaxed ultrafine grains were formed uniformly in both the pin zone (PZs) and the transition zone (TZ-X, Y, Z), with average grain sizes of 450, 410, and 430 nm, respectively, demonstrating the primary feature of the DRX structure. Because of the nature of the DRX structure, the grain sizes were much larger than those obtained by ECAP [116] and HPT [117].

**Figure 16:**
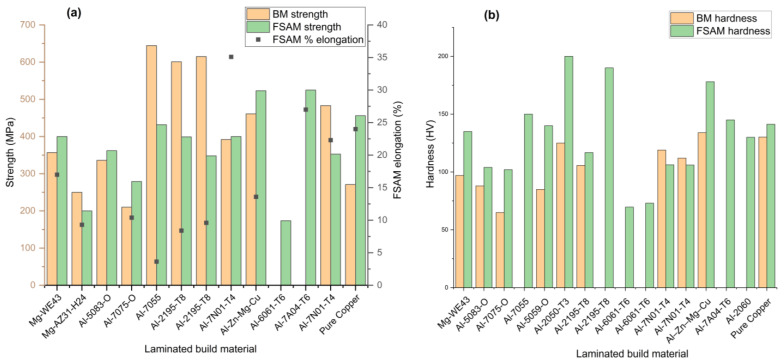
Overall mechanical properties summary of identical material laminates accomplished by researchers (discussed above): (**a**) maximum strength and corresponding elongation (%); (**b**) maximum microhardness.

In conclusion, all the identical material laminates have been discussed in the above section. To get a clear summary of the discussion, the discussed mechanical properties, (tensile strength, elongation and micro-hardness) are also presented graphically in Figure 16.

### 5.2. Multi Material Laminates

#### 5.2.1. Fully Gradient Structure

**Figure 17:**
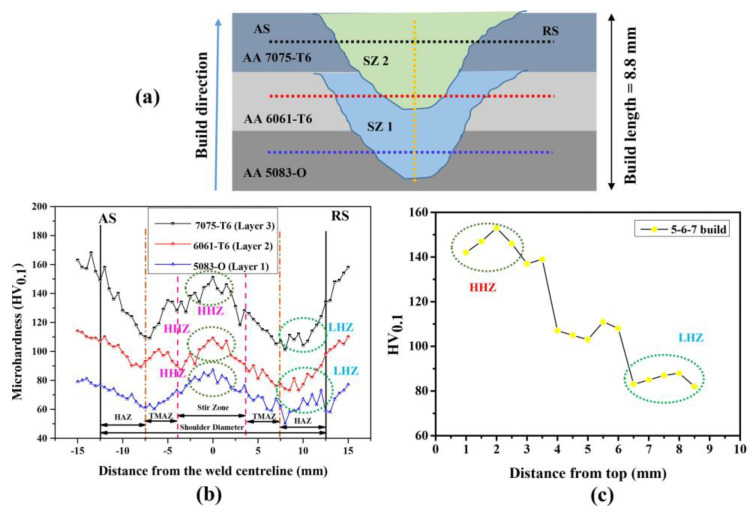
Microhardness variation of a fully gradient laminate: (**a**) complete build with plate order; (**b**) microhardness variation of NZ from the center; (**c**) NZ hardness variation with respect to the building height. (reprinted from [92] with permission from Elsevier).

K. KuFmar [92] fabricated aluminum alloy composite from three different series of aluminum alloys: Al-5083-O, Al-6061-T6, and Al-7075-T6. Plates were stacked in the order of 5, 6, and 7 aluminum series using an optimized taper threaded tool and parameters of 750 rpm and 55 mm/min. Similar to the other researchers’ claims, the hardness value in the stir zone decreased from top to bottom, with the SZ having the highest hardness compared to the HZ and TMAZ. The retreating side (RS) had a low hardness zone, while the TMAZ had a fluctuating hardness value, possibly due to the annealing effect. Results are contrasted below in the Figure 17. Similarly, S. Kumar and A. Srivastava [96] used 3 mm thick plates of Mg-AZ91, Cu, and Al-7075 in order to obtain a fully gradient composite (Al-Cu-Mg) structure through FSAM. To investigate the mechanical properties of the build, a cylindrical threaded tool with a zero-title angle was used at a constant rotation speed of 2000 rpm and a transverse speed of 40 mm/min. The microhardness of each layer in the build direction was reported to be greater than the base metal, with a total of 65.83 HV found in the build.

#### 5.2.2. Alternative Gradient Structure

**Figure 18:**
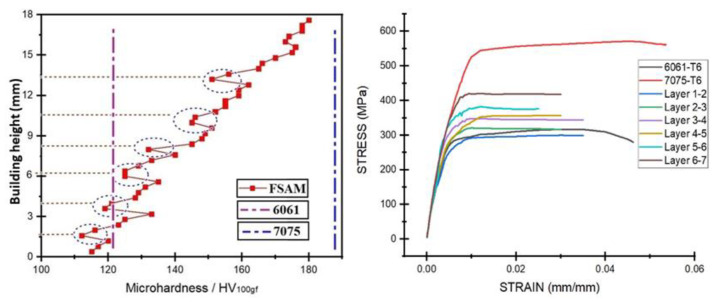
Microhardness and strength illustration of an alternative gradient structure obtained using the FSAM technique (reprinted from [93] permission not required).

H. Venkit [93] created a metallic gradient composite structure by alternately stacking 3 mm thick seven plates of Al-6061-T6 and Al-7075-T6, like the K. Jha [94] study. When the Al-7075 plate was on top, the parameters were set to 1100 rpm and 50 mm/min, and when the Al-6061 plate was on top, the parameters were set to 1200 rpm and 40 mm/min. Because of the different chemical composition, two separate conical threaded pin tools were used, as well as a constant tilt angle of two degrees. Mechanical properties and microstructure were investigated, and it was discovered that hardness and ultimate tensile strength increase from bottom to top (represented in Figure 18), whereas ductility shows an inverse trend similar to the homogenous material laminate developed by M. Yuqing et al. [70], and alternative gradient laminated developed by K. Kumar [92]. The final composite build has greater hardness and ultimate tensile strength than the base Al-6061-T6, but less than Al-7075-T6. The maximum hardness, strength, and elongation were found to be 180 HV, 400 MPa 4.3% in the topmost layer, respectively, with a minimum of 1.7 µm grain size. Due to dynamic recrystallization, uniform material mixing of dissimilar metals with equiaxed grain structure was observed. The use of dissimilar materials also helped to form banded structures.

H. Derazkola [75] employed feed FSAM to acquire a multi-layered polymer-steel composite (poly-methyl-methacrylate-PMMA and S304-textile steel) for automobile application. Eight sheets alternately stacked together to evaluate mechanical properties such as flexural bending, hardness, and tensile strength were evaluated. The hardness in the stir zone (SZ) was lower than on the retreating side (RS), but higher on the advancing side (AS). With some fluctuations, the hardness value increases from top to bottom. Tensile strength is less durable than flexural bending. Because of the lower contribution of textile steel particles in the intermixing with PMMA, the overall targeted mechanical properties were lower than the base PMMA sheet. Later in the study [88], the same technique and method were applied to polypropylene (PP) and textile stainless steel, and it was reported that the flexural and tensile strength was greater than the base PP material, while the hardness and ductility were lower. The incompatibility of the melting temperatures of both sandwiching materials causes hardness reduction in polymer metal composites.

#### 5.2.3. Sandwiched Structure

Z. Tan [72] conducted a numerical and experimental study on aluminum matrix composites reinforced with varying sizes of Al_2_O_3_ nanoparticles. Al-6061-T6 plates with varying width grooves were used. The experiment involved piling the plates together using friction stir welding at a speed of 1000 rpm and 100 mm/min transverse speed. The numerical and experimental results were very close to each other and showed that the particle size of the reinforcing powder and the groove size had a significant impact on microhardness. The findings indicated that a decrease in the Al_2_O_3_ grain size, increase in groove width (resulting in increased volume fraction), and re-stirring led to a reduction in nano-particle grain size, resulting in a high hardness value. Similarly, S. Yan et al. [80] created metal matrix composite (MMC) with ceramic particles of magnesium diboride (MgB_2_) and silicon carbide (SiC) as reinforcing material to enhance space environmental hazards safety. The results show that composites with a high fraction of ceramic particles (more than 30%) obtained via FSAM have a high hardness of 180 HV (approximately twice than cold spray and stir casting) and wear resistance.

M. Roodgari [74] fabricated a composite of interstitial-free (IF) steel and St52 steel using FSAM. The process resulted in sharp interfaces and diffusion in the nugget zone, yielding a hardness of 225 HV and an ultimate tensile strength of 472 MPa, which was higher than the base IF steel but lower than St52 steel. Similarly, Y. Geu et al. [81] created a bimetallic composite of copper and steel using FSAM, which resulted in a periodic wavy structure formation at the interfaces. This method significantly improved the tensile and shear strength compared to other fabrication techniques such as rolling, laser cladding, hot isostatic pressing (HIP), and explosive welding (EXW).

**Figure 19:**
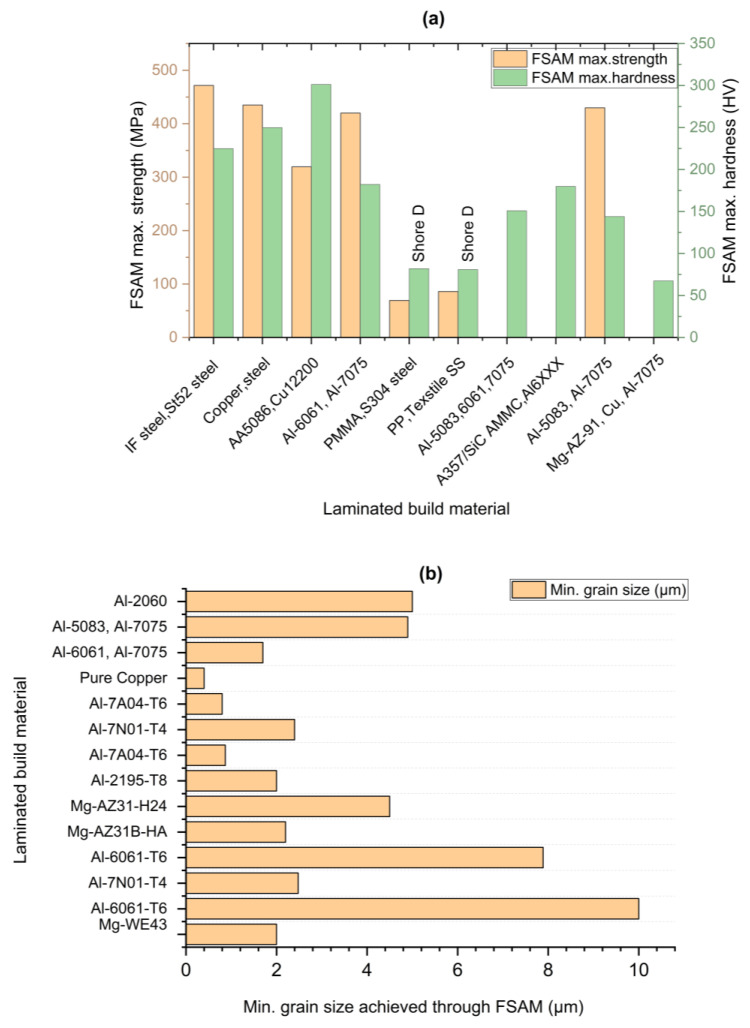
Overall mechanical properties summary (discussed above): (**a**) maximum strength and hardness of multi-material laminates; (**b**) minimum grain size achieved (both identical and multi-material laminates.

In conclusion, all the muti-material laminates have been discussed in the above section. To get a clear summary of the discussion, the discussed mechanical properties, (tensile strength and micro-hardness) and minimum grain size achieved are also presented graphically in Figure 19.

## 6. Viability and Potential Applications of FSAM

Material with ultrafine grain structure (UFG) in bulk form, on the other hand, is gaining popularity due to its surprising high strength and hardness [118,119]. The sever plastic deformation (SPD) method, equal channel angular pressing (ECAP) [120], and high-pressure torsion (HPT) [121] methods are widely used for this purpose. The main issue with these methods is their low ductility, unstable microstructure, and inability to scale up production, which limits their industrial application [122,123]. Thus, as a result of the dynamic recrystallization, FSAM can produce a more stable and uniform microstructure than the conventional SPD process [89]. So, the first question that comes to mind is whether this technique can produce simpler 3D parts with minimal post-processing, or if this technique is only suitable for producing bulk SPD material.

### 6.1. Viability of 3D Part Fabrication

**Figure 20:**
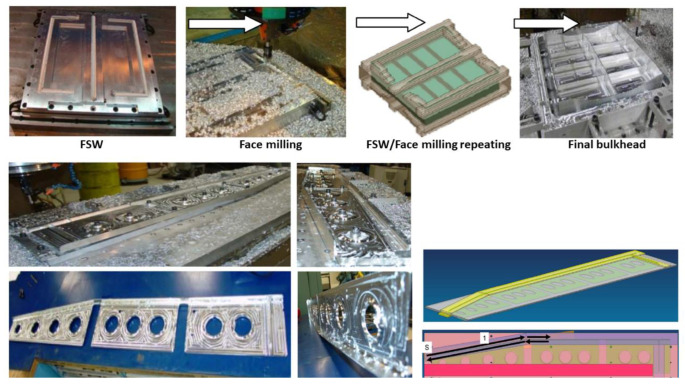
The Production of Energy Efficient Preform Structures (PEEPS); Pseudo Bulkhead and Aircraft 777 floor components (reprinted from technical report [47] permission not required).

Although, FSAM is still in the laboratory phase as a developing AM technique, but some practically near-net shaped prototypes produced through this process reported in the open literature are discussed below. However, no information regarding the results has been provided. The Boeing company [47] collaborated with The Welding Institute (TWI) United Kingdom to produce a near-net shaped structural efficient part of an aircraft 777 with a low buy-to-fly ratio (BTF), as shown below in Figure 20.

**Figure 21:**
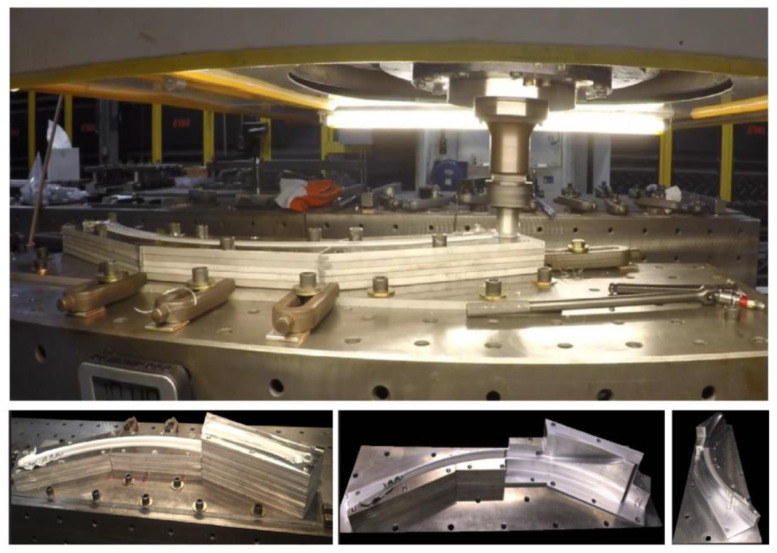
Experimental setup and complex part produced through FSAM by Edison Welding Institute (EWI) (© EWI. reprinted from [125,126] with permission from EWI).

At the 11th World Conference on Titanium in 2007, PL Threadgill and MJ Russell [124] from The Welding Institute UK presented FSAM as a promising technique for aerospace grade titanium alloy (Ti-6Al-4V) to meet the challenge of complex-shaped parts. Similarly, James Cruz [125], manager of the Edison Welding Institute (EWI), skillfully built a large-scale near net shaped component of non-fusionable welded aluminum alloy, and explained it in the article “Does Friction Stir Welding Add Up?”. Experimental setup and part fabrication is shown below in Figure 21.

### 6.2. Potential Practical Application of FSAM

The aerospace industry requires lightweight, high-efficiency structural components. The nut-bolt assembly increases overall weight, and another significant issue is the weldability of aerospace aluminum alloy using conventional welding techniques. At the moment, the parts are made using a subtractive manufacturing process. This entails beginning with plate metal with a thickness equal to the overall part thickness. The material is then machined away in order to create the desired shape or ribbed surface. Ninety percent of the starting martial could be machined away using the process to create the final part. This results in high machining costs and a large amount of material waste. So, FSAM opens avenue for production of such parts, which are discussed blow.

**Figure 22:**
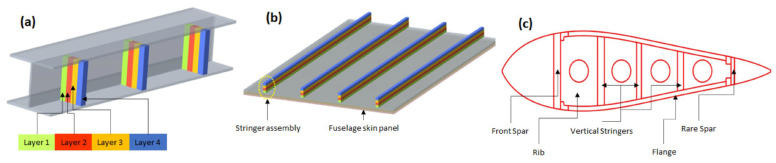
Stiffener/stringer assembly fabrication through FSAM: (**a**) I-beam; (**b**) Fuselage; (**c**) Air foil cross-section.

Potential application I: This technique can be extended to design and manufacture stiffer assemblies such as ribs, fuselage stringers, I beams, and longerons skin panels in the aerospace sectors, as shown in Figure 22 [48].

**Figure 23:**
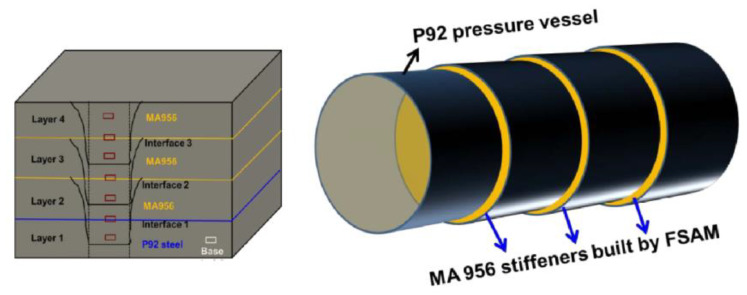
Schematic illustration of MA956 stiffener rings on P92 steel pressure vessel for creep resistance enhancement (reprinted from conference presentation [127] permission not required).

Potential application II: In nuclear and fossil sectors, creep resistant structure can be obtained through this technique. The partial and full stiffener rings, which could be welded around pressure vessels and pipes using the FSAM technique, reduce creep failure under high pressure and temperature, suggested by James Withers during US Department of Energy (DOE) workshop on advanced methods for manufacturing (AMM) [127]. Figure 23 depicts a technique that could aid in the reduction in creep failure in pressure vessels.

**Figure 24:**
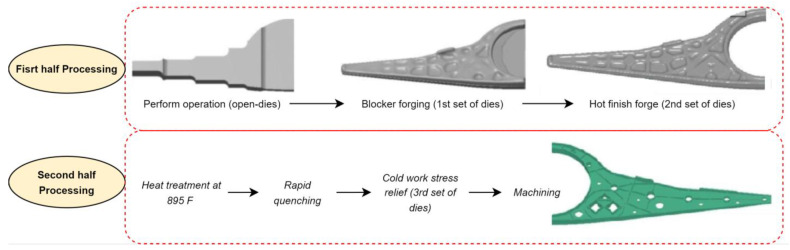
Overview of fictional bulkhead fabrication through existing die forging process followed by Alcoa corporation.

Potential application III: Aluminum bulkheads of fighter jets, which serve as primary support structures, could be manufactured using FSAM. Alcoa corporation currently manufacturing bulkheads using the die forging process is depicted in Figure 24 [128].

**Figure 25:**
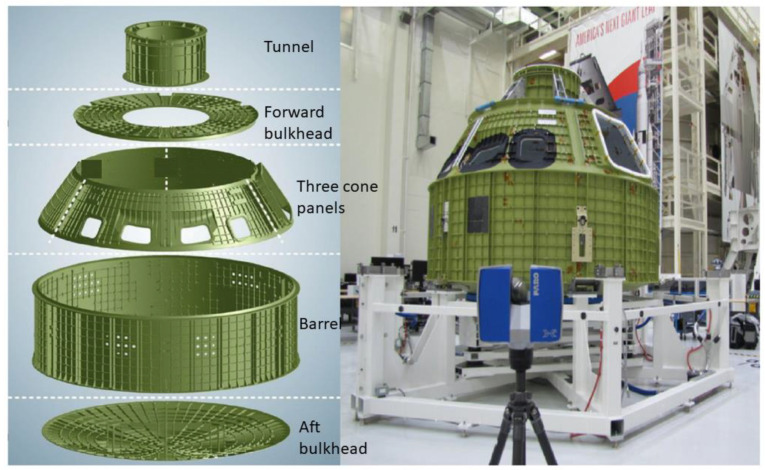
Orion crew’s primary structure and its seven parts which to be welded (NASA) (reprinted from educator guide [129,130] permission not required).

Potential application IV: Various structures of Orion Crew (NASA) are already joined together using friction stir welding [129], so the primary structure of the Orion Crew (NASA) may be constructed using FSAM. Lockheed Martin’s actual Orion exterior crew module has already been 3D scanned, and will also be 3D printed in small scale for the rapid event, according to a press release from Kennedy Space Center in 2016, shown below in Figure 25 [129,130].

## 7. Current Academic Research Status of FSAM

**Figure 26:**
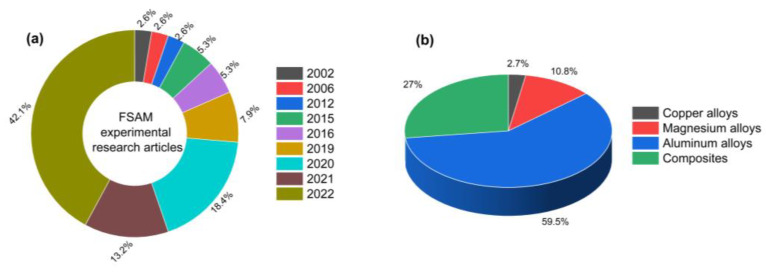
Academic research contribution in FSAM to date (**a**) publication yearly; (**b**) metallic alloy explored.

The experimental academic research contribution to the open literature (until now) on FSAM is presented yearly in the form of a doughnut chart, and the metallic alloys investigated are illustrated in the form of a pie chart in Figure 26 (plotted data is extracted from the literature which is already discussed in the Table 2 and Table 3). According to the doughnut chart, after White filed a patent in 2002 [43], the FSAM was not a noteworthy research topic in academia, with only 2.6% of research publications up to the year of 2012. Following that, research accelerated and gained momentum after 2016, piquing the interest of the research community. The figure shows that research-based studies in the field of FSAM are constantly increasing, with exponential growth year after year. The highest number of research articles published in 2022 is 42.1% of total research publications on FSAM. Similarly, the pie chart depicts the percentage of metallic alloys explored to date in the context of FSAM. According to the graph, copper alloys are the least explored, whereas aluminum alloys are extensively explored as an identical laminate material. Most researchers targeted magnesium alloys (10.8%) and aluminum alloys (59.5%) as a research material in the identical material laminate fabrication, and in the previous couple of years, composites/multi-material laminates have gained interest and contributed 27% of the total.

## 8. Current Technology Readiness Level (TRL) of FSAM

**Figure 27:**
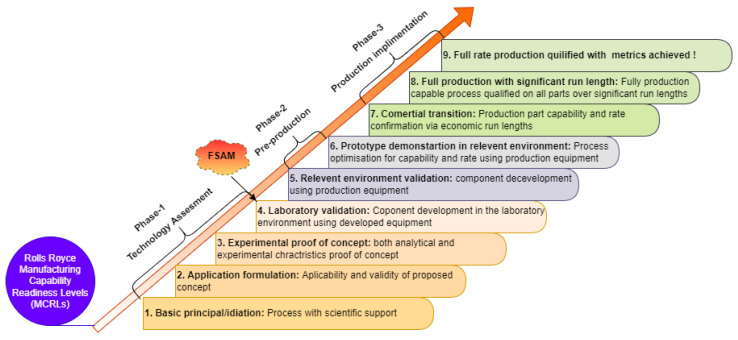
Rolls Royce manufacturing capabilities readiness levels (MCRLs) for TRL identification of FSAM.

Sadin et al. [131] developed technology readiness levels (TRLs) for NASA in 1970 as a method for estimating technology maturity during the program’s acquisition phase, and becoming a more visible component of the development of technologies for future products in the aerospace and defense sectors. Originally, there were seven levels, which J. Mankins [132] updated to give nine TRLs. The U.S department of defense (DoD) is the most active government body in defining a manufacturing readiness standard. Rolls Royce manufacturing capability readiness levels (MCRLs) are now routinely applied throughout the company’s internal and external supply chain and to all sectors of company activity, such as marine, nuclear, and energy applications, and to civil and defense aerospace as well. The basic nine-point MCRL scale is directly derived from TRL, which are split up into three stages: technology assessment, pre-production, and full-scale production implementation. Figure 27 depicts the Rolls Royce MCRL scale and phases. Among these, phase-1 naturally takes a long time to complete and generally covers the entire development process, from concept to demonstration. By the end of phase-1 (at MCRL-4), the fundamentals of process and capabilities ought to be clear [133].

Thus, if above comprehensive review is tied in with the Rolls Royce MRLs to evaluate the development and maturity level of FSAM technology, it can be concluded that FSAM currently lies at MCLR-4 (laboratory validation) and is nearing to transform from laboratory to manufacturing environments. Therefore, it is forecasted that FSAM will become a mainstream additive manufacturing process in the near future.

## 9. Conclusions and Future Recommendations

This novel and advanced additive manufacturing technique (FSAM) was the focus of this critical review. The open literature on process parameters and defects produced, mechanical and microstructural properties, viability, and potential applications, as well as the current academic research status, was investigated and reviewed. The following are the main climactic points of this paper:

- FSAM has been successfully employed to produce defect-free parts with excellent homogeneous mechanical properties, equiaxed refined grain structure, and rapid production rate, thereby addressing the shortcomings of existing melting-based additive manufacturing processes.
- FSAM can also be used to produce bulk material with ultrafine grain structure (less than 0.5 µm).
- Multi-material or new alloys parts with simple geometry can be easily fabricated using FSAM, and parts with a low level of complexity can also be achieved after post processing machining.
- FSAM has gained a lot of attention in the research community in recent years, and it is now at laboratory validation phase (TRL-4) and will soon be used in mainstream additive manufacturing processes.
- The primary challenge of this game-changing process is to eliminate defects by controlling process parameters, and most studies used basic parameters such as rotation speed, transverse speed, and tilt angle. More process parameters, however, must be explored and investigated in order to obtain a sound microstructure and mechanical properties.
- Although mechanical properties such as micro-hardness and tensile strength are extensively studied for various material additive laminates, failure properties under cyclic loading are rare and should be investigated.
- Among non-ferrous alloys, only a few aluminum aerospace alloys (~60%) have been studied in the context of FSAM to date, followed by magnesium-based alloys (~11%). However, other harder alloys such as titanium and nickel alloys need to be further explored in the future in the context of this novel technique.

## Figures and Tables

**Table 1:** Merits and limitations of FSAM over existing meting-based AM [12,54,55,56,57,58].

Merits	Limitations
Homogeneous, equiaxed ultrafine microstructure.	Incompetence to fabricate intricate shapes/complex geometry.
High structural integrity with superior mechanical properties.	Tool wear and workpiece clamping dilemmas.
Solidification defects are negligible.	Considerable residual stresses.
High production rate and volume as no vacuum/inert gas chamber required.	Prior layer flash removing necessary before adding next layer.
Less energy consumption (~2.5% of fusion-based process).	Some post processing needed to obtain net shape.
There is no powder related restriction as feed material is in plate form.	
Smaller heat affected zone (HAZ).	
More sustainable due to fumeless process or very less of greenhouse gases.	
Non-welded high-strength alloys and dissimilar alloys in graded fashion can be processed.	

**Table 5:** Summary of microstructural defects originated in the multi-layered build investigated by researchers.

Material	Parameters			Defect Type					Ref.
	Pin Profile	rpm/mmmin^−1^/Tilt Angle	Medium	Hooking	Kiss Bonding	Cavity	Tunnel/Micro-Voids	Cracking	
WE43 rolled	Right-handed stepped spiral	1400/102/1.5°	Air	✓	—	✓	—	✓	[32]
AA 7075-O	Left cylindrical threaded	600/60/2°	Air	✓	✓	—	—	—	[70]
2050 Al-Cu-Li Alloy	Threaded taper with 3 flats	250/204/1°	water spray	✓	—	✓	—	✓	[71]
Al-Li 2195-T8	Cylindrical and flared pin with 3 concave arc grooves	800, 900, 1000/100	Air	✓	✓	✓	—	—	[62]
AA 7N01-T4	Right-handed stepped spiral	1200/60	Air	✓	✓	—	—	—	[73]
IF, St52 steel	Cylindrical	600/40/3°	Air	—	✓	—	—	—	[74]
Mg alloy AZ31-H24	Threaded taper triangular	>1000/>100/−0.5°	Air	—	—	✓	✓	—	[78]
AA 6061-T651, Steel1018	Cylindrical	1000/300/1°	Air	—	—	—	✓	✓	[82]
AA5086 and C12200	Cylindrical	400, 500, 700/40, 60, 80/3°	Air	—	—	✓	✓	—	[90]
Al 5059-O	Taper threaded	450/63/2°	Air	✓	—	✓	—	—	[91]
Al-5083-O,6061-T6, 7075-T6	Taper threaded	750/55/3°	Air	✓	✓	—	—	—	[92]
Al-6061, Al-7075	Taper threaded	1100, 1200 /40, 50/2°	Air	✓	✓	—	—	—	[93]
Al-7075-T6	Threaded taper with 3 flats	2000/65, 80, 95/0.5°	Air	✓	✓	—	—	—	[67]

## Data Availability

No new data were created or analyzed in this study. Data sharing is not applicable to this article.
